# Metallic and Metal Oxides Nanoparticles for Sensing Food Pathogens—An Overview of Recent Findings and Future Prospects

**DOI:** 10.3390/ma15155374

**Published:** 2022-08-04

**Authors:** Camelia Ungureanu, Gratiela Teodora Tihan, Roxana Gabriela Zgârian, Irina Fierascu, Anda Maria Baroi, Silviu Răileanu, Radu Claudiu Fierăscu

**Affiliations:** 1 Department of General Chemistry, University “Politehnica” of Bucharest, 011061 Bucharest, Romania; 2 National Institute for Research & Development in Chemistry and Petrochemistry ICECHIM, 060021 Bucharest, Romania; 3 Faculty of Horticulture, University of Agronomic Sciences and Veterinary Medicine of Bucharest, 011464 Bucharest, Romania; 4 Department o of Automation and Industrial Informatics, University “Politehnica” of Bucharest, 011061 Bucharest, Romania; 5 Department of Science and Engineering of Oxide Materials and Nanomaterials, University “Politehnica” of Bucharest, 011061 Bucharest, Romania

**Keywords:** metallic nanoparticles, metallic oxides, biosensors, food-borne pathogens, nanotechnology, rapid detection

## Abstract

Nowadays, special importance is given to quality control and food safety. Food quality currently creates significant problems for the industry and implicitly for consumers and society. The effects materialize in economic losses, alterations of the quality and organoleptic properties of the commercial products, and, last but not least, they constitute risk factors for the consumer’s health. In this context, the development of analytical systems for the rapid determination of the sanitary quality of food products by detecting possible pathogenic microorganisms (such as *Escherichia coli* or *Salmonella* due to the important digestive disorders that they can cause in many consumers) is of major importance. Using efficient and environmentally friendly detection systems for identification of various pathogens that modify food matrices and turn them into food waste faster will also improve agri-food quality throughout the food chain. This paper reviews the use of metal nanoparticles used to obtain bio nanosensors for the purpose mentioned above. Metallic nanoparticles (Au, Ag, etc.) and their oxides can be synthesized by several methods, such as chemical, physical, physico-chemical, and biological, each bringing advantages and disadvantages in their use for developing nanosensors. In the “green chemistry” approach, a particular importance is given to the metal nanoparticles obtained by phytosynthesis. This method can lead to the development of good quality nanoparticles, at the same time being able to use secondary metabolites from vegetal wastes, as such providing a circular economy character. Considering these aspects, the use of phytosynthesized nanoparticles in other biosensing applications is also presented as a glimpse of their potential, which should be further explored.

## 1. Introduction

**Figure 1:**
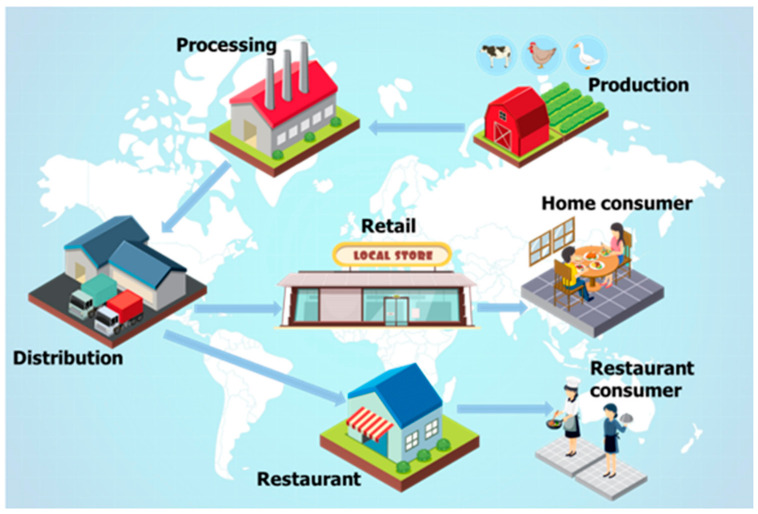
Food contamination chain (all graphical illustrations were created using materials from the free images database: www.freepik.com).

The world’s growing population and limited natural resources are closely linked to agricultural activity, and therefore, to productivity growth, which must take into consideration food security. As a result of globalization, the industrial agricultural practices evolved, increasing food production, but at the same time, the potential risk of food contamination increased. According to the World Health Organization (WHO), billions of people in the world are at risk, and millions become sick after consuming contaminated food every year [1]; therefore, it is very important to develop quick, specific, sensitive, and cost-effective methods to detect food contaminants. Our lives depend on how we choose to live, so many of us are thinking lately about adopting a healthy lifestyle. In addition to rest and movement, nutrition is an extremely important factor in the harmonious development of the human being. We need to look for reliable and safe foods with no contaminants. Contaminated food refers to the presence of potential harmful chemicals and/or micro-organisms, which can affect food and therefore cause illness to consumers [2]. Contamination can occur throughout the supply chain, from the producer to the consumer (Figure 1) [3].

Similar definitions of food are given by the Cambridge dictionary (“something that people and animals eat, or plants absorb, to keep them alive”) and the Oxford dictionary (“any nutritious substance that people or animals eat or drink or that plant can absorb in order to maintain life and growth”) [4,5].

In terms of food safety, preventing contamination is the first approach, but despite careful handling, food can be contaminated by pathogens. Therefore, food nanotechnologies are necessary for food safety and analysis. Among other fields, the food industry is about to be revolutionized by nanotechnology. It seems that “nanoeating” represents a whole set of scientific ideas that are already being implemented and applied in the industry [6]. First, nanotechnology can provide food producers with unique opportunities for full real-time monitoring of product quality and safety in the production process. We are talking about “diagnostic” machines that use various nanosensors or so-called quantum dots (QD) that can quickly and reliably detect the smallest chemical contaminants or biological hazardous agents in products [7].

There are many terms correlated with nanotechnology, but two of the most used expressions are “nanomaterial” and “nanoparticle”. While sometimes both expressions are used interchangeably, the American Society for Testing and Materials (ASTM) [8] states that the nanoparticle (NP) is a subclassification of a “two- or three-dimensional ultrafine particle larger than 1 nm and smaller than about 100 nm which may or may not have a size-intensive property”. However, then, the European Union has defined nanomaterial (NM) as a “natural, incidental or manufactured material containing particles in an unbound state or in aggregate or agglomerated form, for which more than 50% of the dimensional distribution of the particles, of one or more dimensions, is in the range 1–100 nm” [9].

**Figure 2:**
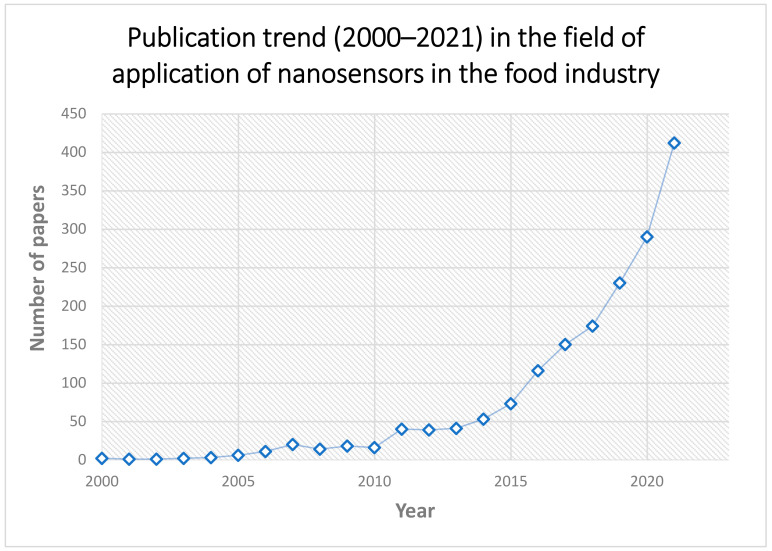
Publication trend (2000–2021) in the field of application of nanosensors in the food industry (source of raw data: https://www.sciencedirect.com; search keywords: nanoparticle, food, nanosensors).

Over time, for the detection and identification of food contaminants, various analytical procedures were developed [10,11], using sophisticated instrumental techniques, such as high-performance liquid chromatography–mass spectrometry (HPLC-MS), polymerase chain reaction (PCR) [12,13], enzyme-linked immunosorbent assay (ELISA) [14]. Considering that tests such as PCR and ELISA are destructive, time consuming, require well-trained specialists, and could produce erroneous results due to the presence of inhibitors in food, new methods are constantly researched. The sensor/biosensor-based nanotechnology is a state of the art for extensive applications in the detection of pathogenic bacteria, as they are self-contained, low cost, with minimal power requirements [15]. Figure 2 emphasizes an annual increase rate of publications, with an accelerating trend from 2016 to the present (the graphs only present works published up to the end of 2021). Compared to other papers, their number is not very big, and for this reason, this manuscript is important to focus the researchers’ attention on the usage of metallic and metal oxide nanoparticles for sensing food pathogens, considering their properties.

The present review aims to be a critical discussion regarding the use of metallic and metal oxide nanoparticles for sensing food pathogens. It contains general information regarding the different methods used for the synthesis of metallic nanoparticles (physical, chemical, physico-chemical, and biological methods being presented), the types of bio-nanosensors for food applications, application of metallic nanoparticles for sensing food pathogens, safety and regulatory issue, conclusions, and future perspectives. The review also contains a chapter presenting the application of phytosynthesized nanoparticles as biosensors, as an example of the potential application of this particular type of NPs, which could become a viable “greener” alternative for food pathogens sensing.

## 2. Synthesis of Metallic Nanoparticles

One of the main limitations regarding the use of NPs in a sensing application is related to the lack of a synthesis method that can, at the same time, lead to controlled size and morphology NPs, homogeneous in nature and with a narrow size distribution, with little or no toxicity, without using any potentially toxic compounds or hazardous techniques [16]. From the multitude of synthesis methods, each with its advantages and disadvantages, the appropriate one must be selected for developing NPs with the desired size and morphology, as well as successful scale-up for industrial application. According to several publications [17,18], the development of synthesis methods of nanoparticles beyond the laboratory scale should take into account the clarification of issues related to: production quantification of nanomaterials/nanoparticles, establishment of a production time for a certain volume of nanoparticles, standardization of the conditions for obtaining specific monodisperse particles and their stability. The most used metals are Au, Ag, Pt, Pd, Zn, Cd, Cu, and Fe, while the precursors that can be used are represented by the corresponding metallic salts, such as HAuCl_4_, AgNO_3_, H_2_PtCl_6_, PdCl_2_, Zn(CH_3_COO)_2_, CdSO_4_, CuSO_4_, or Fe(NO_3_)_3_ [19]. 

The synthesis of metallic nanoparticles can be achieved by several methods:

(1) physical methods based on obtaining particles by physical exposure (laser ablation, dispersion, evaporation/condensation, etc.);
(2) chemical techniques in which the process of particle synthesis is initiated by chemical action;
(3) combined methods of obtaining particles (physico-chemical);
(4) biological methods based on bioreduction using plant extracts and bioreduction using microorganisms.

### 2.1. Physical Methods

#### 2.1.1. Method of Mechanical Dispersion Using BEAD Mill

There is a known method in which it is possible to obtain the highest degree of dispersion when using colloidal mills, whose principle is based on creating sufficiently large centrifugal forces in a narrow space between a rotating rotor and a stationary stator, leading to mechanical breaking of larger particles found in suspensions or emulsions [20]. The simplest method of dispersion is to use a ball mill or mortar. These methods, in principle, do not allow one to obtain truly colloidal systems because, along with the dispersion process, the opposite process associated with the expansion of particles—an aggregation process—is always present. The positive side of mechanical grinding methods is the simplicity of installations and technologies, the ability to grind various materials, as well as the ability to obtain material in large quantities. Disadvantages of the method include the possibility of contamination of powders obtained with abrasive materials, the difficulty of obtaining materials with controlled morphology, the difficulties in controlling the composition of the product in the grinding process, and the crystal defects induced by the method [21]. Takashi Ogi et al. [21] highlighted the importance of these methods for obtaining different types of nanomaterials, such as TiO_2_ nanoparticles. 

#### 2.1.2. Plasma Method

Another method utilized for the synthesis of nanoparticles is the plasma method. In this case, plasma is produced by the radio frequency (RF) heating coil. The most used plasma processes are microwave and plasma-spray synthesis [19]. A thermal plasma can also provide the energy needed to cause the micrometer-sized particles to evaporate. Thermal plasma temperatures are around 10,000 K; as such, the solid dust evaporates easily. Nanoparticles form in the cooling phase, while the plasma state exists. In RF induction plasma torches, the energy associated with the plasma is realized by the electromagnetic field generated by the induction coil. Because the residence time of food droplets injected into the plasma is very short, it is necessary to use droplets small enough, so that complete evaporation can be achieved [22]. Other disadvantages of the method include the high costs of equipment (although the nanomaterials’ production costs are overall relatively reduced) and limitations regarding the possibilities of obtaining more complex structures of precise morphologies [23].

#### 2.1.3. The Aerosol Technique

Aerosol-based processes are mainly used for the application of coatings. Chemical precursors sprayed on a surface in a heated medium form nanoparticles after being subjected to pyrolysis [24]. The method leads to narrow-size distribution NPs and allows the synthesis of complex structures, although, as in the plasma method, the method involves expensive equipment. 

#### 2.1.4. Laser Ablation

Laser ablation is a method for fabricating various types of nanoparticles. With laser ablation, using a high-power laser beam with an optical focusing system and a target feeding system, nanoparticles can be obtained [25]. The characteristics of the nanoparticles produced by laser ablation depend on many factors: the wavelength of the laser influencing the metal charge, the duration of the laser pulsations (in femto-, pico-, nanoseconds), the laser flux, the ablation time, and the effectiveness of the liquid medium, in the absence or presence of surfactants. The efficiency of femto-second ablation in water was found to be lower than that in air, while in the case of nanosecond pulsations, the efficiency of ablation was similar in both water and air [26]. An important advantage of the laser ablation technique, compared to other methods, is represented by the lack of any chemical reagents necessary for obtaining the NPs. As such, using this method can result in pure and uncontaminated colloidal metals and can also achieve the in situ conjugation with biomolecules; a major disadvantage of the method is represented by the high costs of the equipment necessary, as well as the energy consumption of the method and the overall low yield [27]. Using this method, different types of NPs can be obtained, and their properties can be tuned for specific applications. For example, magnetite nanoparticles were obtained by Svetlichnyi et al. [28] using a Nd:YAG laser, which can be further transformed into other types of iron oxide NPs via thermal treatment.

#### 2.1.5. The Gas-Phase Synthesis

Metallic nanoparticles can also be prepared by gas-phase synthesis. The gas-phase condensation process is the most used method of producing metal or metal oxide nanoparticles. The main parts of the condensing system are a vacuum chamber connected to a heating element, the material to be vaporized, a cooling chamber connected to a dust collection system, and a pumping system. The material in the vacuum chamber is brought to a temperature and pressure to establish a flow of matter, which collides with the gas present in the cooling chamber, forming spherical nanoparticles. Depending on the application, the coolant may be reactive or inert [29]. Inert gas condensation is commonly used to form nanoparticles of metals that have a low melting point. Super-cooled metal vapors condense into nanometer-sized particles, which can be suspended in a stream of inert gas and stored on a substrate or studied in situ [30]. The method leads to the synthesis of high-purity nanoparticles, although obtaining more complex materials or materials with higher melting points represents its main drawbacks [31]. 

#### 2.1.6. Spark Discharge

The technique is based on the electric charge of two electrodes composed of the metal to be vaporized. By exploiting the phenomenon of the electric arc, it is possible to vaporize small amounts of metal sufficient to form nanoparticles. Often, spark ionization is classified as an arc discharge, even though the two methods are slightly different; in the arc method, the discharge is continuous, while in spark ionization, it is momentary [20,21]. The application of the method results in high-quality nanoparticles and, not considering the initial costs of the equipment, is reasonably cost effective.

#### 2.1.7. Sonochemical Reduction

In the case of sonochemical reduction, the chemical effect of ultrasound occurs due to acoustic cavitation, consisting of the formation, growth, and implosion of bubbles in the liquid. The implosion collapse generates a local hot spot by adiabatic compression or by the formation of shock waves in the gas phase inside the collapsing bubble [22,23,24]. The method can be applied to synthesize several types of NPs, a recent review work presenting the synthesis of Fe_3_O_4_, Au, and Fe_3_O_4_/Au NPs [32]. Although the method has several advantages (including the reduced reaction time and controlled morphologies), the main drawback of the method is represented by the difficulties in the scale-up for industrial application and its cost effectiveness [33].

#### 2.1.8. Gamma-Ray Radiation

Gamma-ray radiation is a physical method used for metallic nanoparticles synthesis [34]. Thi Kim Lan Nguyen and coworkers [35] reported the synthesis of platinum nanoparticles by gamma-ray radiation using chitosan as a stabilizer, while Duy Khang Nguyen Vu et al. successfully synthesized a silver nanoparticle embedded in graphene oxide/TiO_2_ nanotube nanocomposite using this method [36]. Different types of metal and metallic oxides NPs can be obtained by this method, representing one of the “green” synthesis methods. Although the method allows the synthesis of NPs of controlled size and morphologies, the main drawback of the method is represented by the strict security measures necessary, as the use of ionizing radiation can raise several issues regarding the associated health hazards. Additionally, the method needs the presence of a capping agent (acting as a stabilizer), usually polyols [37].

#### 2.1.9. Microwave Irradiation

Microwave irradiation is another method for the one-pot synthesis of metallic nanoparticles in solutions. Morphologies and sizes of metallic nanoparticles could be controlled by changing the experimental parameters, such as the concentration of metallic salt, the type of solvent and surfactant, and the reaction temperature. Although the method has several advantages (including shorter reaction times compared with other methods, higher yields, or very good reproducibility), it also has some major drawbacks, such as the relative high costs of the equipment and difficulties in the scale-up or reaction monitoring [38].

Masaharu Tsuji et al. obtained Ag, Au, Pt, and AuPd nanoparticles using this method [39]. The same method was applied for the synthesis of Au or MoO_2_ NPs [40,41].

### 2.2. Chemical Methods

Chemical methods represent the most common approach for the synthesis of nanoparticles. This involves a chemical reduction in the metal precursor using organic or inorganic agents. Various reducing agents, such as Tollens’s reagent, N, N-dimethylformamide (DMF), sodium citrate, ascorbate, and polyethylene glycol (PEG), are used to reduce metallic ions in different solutions. It is important to use stabilizers for dispersed nanoparticles during the synthesis and to protect nanoparticles that may be absorbed or trapped on surfaces, thus avoiding agglomeration [42]. Polymeric compounds (polyvinyl alcohol, polyvinylpyrrolidone, polyethylene glycol, polymethacrylic acid, and polymethyl methacrylate) have been declared as highly effective protecting agents in the nanoparticle stabilization process [43]. However, following all the chemical methods has a common disadvantage, represented by the need for chemical reduction agents or stabilizers, which makes them not the primary choice in the search for “green” synthesis methods [33].

#### 2.2.1. The Preparation of Nanoparticles in Biphasic Aqueous Organic Systems

The preparation of nanoparticles in biphasic aqueous organic systems is based on the spatial separation of the reactants (the precursor and the reducing agent) in two immiscible phases [44]. The biggest disadvantage of this method is the use of very dangerous organic solvents. Large amounts of surfactants and organic solvents must be separated and removed from the final product. A simple and efficient synthesis method would be UV-initiated photoreduction, which is used in the synthesis of metallic nanoparticles in the presence of sodium citrate, polyacrylic acid, or collagen, for example [45]. Huang and Yang (2008) [46] obtained silver nanoparticles by photoreduction in silver nitrate into layered inorganic substances that they used as a stabilizing agent against nanoparticle aggregation [47]. The properties of the obtained nanoparticles were evaluated as a function of UV irradiation time.

#### 2.2.2. Turkevich Method 

The most popular synthesis method of spherical AuNPs is the Turkevich method, which involves reducing HAuCl_4_ with sodium citrate in aqueous solution at boiling point. Using this method, nanoparticles of different sizes, with diameters between 10 and 150 nm, can be obtained by only varying the ratio of the two reactants [48]. The major advantage of the method is the possibility to control the NPs size by controlling the citrate/chloroauric acid molar ratio. The main disadvantage of the method is represented by the very small particle size domain for which the method preserves its reproducibility and ability to be scaled-up (15–30 nm) [48]. 

#### 2.2.3. The Brust–Schiffrin Method

Another method for the synthesis of AuNPs of smaller dimensions is represented by the Brust–Schiffrin method. The very low-dimensions AuNPs (1–3 nm) are obtained in organic liquids in order to ensure the coating, which can control growth rates; using this method, anisotropic metal nanoparticles, such as rods, triangles, stars, or flower-like structures, are obtained [49]. 

Although the methods described above provide high-quality nanoparticles, they use some chemical agents (e.g., sodium citrate, bromides, alkanethiols, etc.) known for potential biological risks, which only have limited stability of AuNPs in biological environments and still require high purification.

#### 2.2.4. The Tollens Method

A direct and simple process for the synthesis of AgNPs is represented by the Tollens method used for the synthesis of size-controlled Ag nanoparticles. This method includes a reduction in the Tollens reagent with an aldehyde [50]. In the modified method, Ag ions are reduced by saccharides in the presence of ammonia, resulting in nanoparticle films of Ag (50–200 nm), colloidal silver (20–50 nm), and nanoparticles of various shapes [51]. Although a modified Tollens method [52] uses saccharides for the reduction process, the presence of ammonia is still necessary; as such, the method does not possess a truly “green” character. More than that, the method can be applied only for the development of silver NPs.

#### 2.2.5. Hot Injection Method

Another chemical method used to obtain metal nanoparticles is called hot injection, and it is a batch process in which chemical precursors are rapidly injected into a heated reactor containing a mixture of solvents and ligands [53]. The method leads to the obtention of NPs with a narrow size distribution, with high crystallinity, and with controlled size and morphology. The main drawbacks of the method are represented by the high associated costs, use of toxic chemicals, and the difficult scale-up. 

Using this method, NPs of different size and morphology can be obtained, including metallic [54], bimetallic [55], or other complex structures (such as CoFe_2_O_4_ [56], FeS_2_ [57], Cu_2_SnSe_3_ [58], or Cu_2_NiSnS_4_ [59]).

#### 2.2.6. Microemulsions Method

Uniform, size-controlled nanoparticles can be obtained using microemulsion techniques [60]. The method is easy to apply, thermodynamically stable, and does not require any energy consumption. Its main disadvantages are the high amounts of surfactants necessary, as well as the multiple factors influencing the process. Monnoyer et al. [61] synthesized nanoparticles of metals, such as Co, Ni, and metal alloys Fe–Ni, Cu–Au, and Co–Ni, using reverse micelles. 

Silver and gold nanoparticles were also synthesized by water-in-oil microemulsion method [62] by reducing metal precursors with citric acid at 80 °C for 30 min. 

Using the microemulsion method, different types of magnetic materials were also obtained, such as FeNi NPs [63] or magnetite [64].

#### 2.2.7. Thermal Decomposition

Thermal decomposition is another route (easy and economical) to synthesize monodispersed metallic nanoparticles, which are stable and of small particle sizes; no stabilizer is used for thermal decomposition [65]. The method leads to monodispersed NPs in relatively high amounts. Drawbacks of the method include the frequent use of surfactants and organic precursors and the high temperature necessary to be maintained for relatively long time periods. 

For example, Unni et al. [66] obtained magnetite NPs by thermal decomposition of the iron oleate precursor at 350 °C for 5 h.

#### 2.2.8. The Polyol Method

The polyol method is an important synthesis route, as it provides a high degree of control over both the size and geometry of the metallic nanoparticles. There are many papers that have reported the synthesis of oxide sub-micrometer particles: Mn_3_O_4_, ZnO, SnO_2_, PbO, or TiO_2_ [67,68]. The method allows the synthesis of controlled size and morphology, although the reaction occurs over long periods of time. Oh et al. [69] obtained different-sized magnetite NPs by varying the molar ratio FeCl_3_/H_2_O, respectively, the sodium acetate concentration.

#### 2.2.9. The Sol-Gel Process

The sol-gel process represents a useful method for the synthesis of many nanomaterials, especially metal oxide NPs. This method of obtaining the metallic nanoparticles is fast, simple, and inexpensive. The sol-gel method presents numerous other advantages, such as homogeneity of the nanoparticles, the high purity of the end product, and the low processing temperature; the disadvantages of the method include the relatively high costs of the organo-metallic precursors, use of some toxic compounds, and the necessity for a post-synthesis calcination step. By this process, metal oxide nanoparticles, such as titanium dioxide, tin oxide, tungsten oxide, or zinc oxide, can be obtained [70,71].

#### 2.2.10. Chemical Vapor Deposition (CVD)

Chemical vapor deposition involves a chemical reaction and one or more volatile precursors. The method allows the synthesis of high-purity and homogeneous NPs but is strongly dependent on the substrate; high temperatures are necessary, and the by-products are represented by toxic gases [33]. Using this method, NPs such as TiO_2_, ZnO, NiO, CoFe_2_O_4_, etc., can be obtained [72,73,74].

### 2.3. Physico-Chemical Methods

#### 2.3.1. Electrochemical Synthesis

Electrochemical deposition represents a process in which the NPs are obtained at the interface metal containing electrolyte/electrically conductive metal substrate [75]. Using this method, it is possible to control the size of the metal nanoparticles and their morphologies (nanorods, nanowires, nanotubes, nanosheets, composite nanostructures being obtained [76]). The method has several advantages, including the low costs and temperatures associated, simplicity, and the high purity of the obtained materials. The main drawback of the method is related to the use of stabilizing agents, a step that must be optimized for each type of NP and which can affect the real-life application of the method. 

For example, silver nanodendrite NPs were obtained by an electrochemical technique, using pure metallic silver as an electrode, glassy carbon rod as a counter electrode, and an electrolyte prepared using deionized water [75].

#### 2.3.2. Microwave-Assisted Chemical Synthesis

Microwave-assisted chemical synthesis is another method for obtaining metallic nanoparticles, using the advantages of the previously presented microwave synthesis and chemical synthesis. It has been reported, for example, that Ag nanoparticles were obtained by this method, involving sodium carboxymethylcellulose as a reducing and stabilizing agent. The size of the resulting particles depends on the concentration of sodium carboxymethylcellulose and on the silver nitrate concentration. The nanoparticles produced are uniform and stable [77]. The production of Ag nanoparticles in the presence of platinum (Pt) granules, polyvinylpyrrolidone, and ethylene glycol has also been reported. In addition, starch was introduced as a template and as a reducing agent for the synthesis of Ag nanoparticles with an average size of 12 nm using the microwave-assisted method. The function of starch (as a template) is to prevent the aggregation of the produced AgNPs.

### 2.4. Biological Methods

#### 2.4.1. Bioreduction Using Plant Extracts

In recent years, plant extracts have been used for the synthesis of metal nanoparticles, thus offering numerous benefits, especially in biomedical, pharmaceutical, and food applications, the nanoparticles and the natural compounds potentiating each other’s beneficial effects. Herbal extracts are one of the most studied categories today and are considered one of the most promising sources of natural reducing agents. The compounds in the extracts act both as reducing agents as well as stabilizers for the developed metal nanoparticles [78,79]; as such, practically any type of NP can be obtained by this method. 

The research area regarding nanoparticles obtained by this green synthesis method should consider the identification of plant-derived materials most suitable for the synthesis of specific nanomaterials/nanoparticles, as well as the clarification of the biochemical and molecular mechanisms involved in the formation of specific nanoparticles. Green nanotechnology, resulting from the combination of nanotechnology with the principles and practices of green chemistry, constitutes an important element in the development of an environmentally sustainable society in the 21st century. Current processes in the area of eco-nanotechnology involve the use of natural sources, non-toxic solvents, and energy-efficient processes for nanomaterials development [80,81,82]. 

Phytosynthesis of metallic nanoparticles (using vegetal material extracts) has, as a first step, the obtention of the extract, which can be achieved by extraction methods with classic (Soxhlet extraction, percolation, etc.) or modern instruments, such as microwave-assisted extraction. The factors influencing the morphological characteristics of silver nanoparticles are numerous, including the plant characteristics, the plant parts used, the extraction method, the solvent used, salt concentration, and other synthesis conditions [37,83,84,85]. 

Our research group evaluated in vivo and in vitro the silver nanoparticles phytosynthesized using *Raphanus sativus* L. waste extracts [86], gold and silver nanoparticles obtained using *Aconitum toxicum* Reichenb. rhizomes alcoholic extracts [87], metallic nano-architectures developed via *Melissa officinalis* L. extracts [88], as well as compared the efficiency of phytosynthesized and radiation-assisted synthesized metallic nanoparticles [37].

*Camellia sinensis* (L.) Kuntze extract (green tea) has been used as a reducing and stabilizing agent for the biosynthesis of Ag nanoparticles in aqueous solutions and under ambient conditions [89]. It was observed that when the amount of *C. sinensis* extract increased, the resulting nanoparticles were relatively larger, wider, and more spherical. The phenolic acid biomolecules (e.g., caffeine, theophylline) present in the *C. sinensis* extract are responsible for the formation and stabilization of silver nanoparticles. It has also been reported that metallic nanoparticles were obtained from plants, including: dill, sage, orange, and grapefruit wastes, burdock, southernwood, asparagus, tangerine, *Urtica dioica* leaves, and *Vitis vinifera* fruits’ extracts, weed herb *Cirsium arvense*, or leaves and rhizomes of *Asplenium scolopendrium* L. [90,91,92,93,94,95,96]. 

Naseer and coworkers [97] reported the eco-synthesis of platinum nanoparticles using the plants’ aqueous extracts, while Velmurugan et al. [98] presented the synthesis of platinum nanoparticles mediated by *Prunus × yedoensis* Matsum. extract.

Additionally, the phytosynthesis of 20 to 30 nm magnetic magnetite nanoparticles with diverse morphologies was demonstrated by Geneti et al. [99] using *Thymus schimperi* leaves extract. The authors proposed the application of the developed NPs for metal removal from contaminated water.

Although the method has many advantages and can provide a true “green” character to the synthesis, it also has several disadvantages, including the difficulties in controlling the size and morphologies of the NPs and the incompletely elucidated synthesis mechanisms.

#### 2.4.2. Bioreduction Using Microorganisms

Many studies have been performed on the synthesis of Ag nanoparticles using organisms (microorganisms and biological systems) [100,101,102,103]. For example, the bioreductive synthesis of silver nanoparticles using the fungus *Fusarium oxysporum* has been demonstrated [101,104]. Very stable Ag nanoparticles (40 nm) can also be synthesized by bioreducing Ag (aq) ions using a culture of non-pathogenic bacteria, *Bacillus licheniformis* [105]. In addition, well-dispersed Ag nanocrystals (50 nm) were synthesized using this bacterium. Saifuddin and his team [106] have discovered a new method for nanoparticle synthesis. This approach is achieved by combining supernatant cultures (*Bacillus subtilis*) with the method of microwave irradiation in water. They described the extracellular biosynthesis of Ag nanoparticles using *Bacillus subtilis*, but instead of increasing the reaction rate and reducing the aggregation of the nanoparticles, they used microwave radiation, which helps to evenly heat the nanoparticles.

Ag nanoparticles (5–50 nm) can be synthesized extracellularly using *Fusarium oxysporum*, with no evidence of particle flocculation even one month after the reaction. The long-term stability of the solution given by the nanoparticles may be due to the stabilization of Ag nanoparticles by proteins. The morphology of the nanoparticles varies with the shape, being generally spherical, occasionally with triangular shapes being present [104]. Stable Ag nanoparticles can also be obtained using *Aspergillus flavus*. These nanoparticles are stable in water for more than 3 months without any significant aggregation due to the surface binder of the stabilized materials secreted by fungi [103]. Extracellular biosynthesis of Ag nanoparticles using *Aspergillus fumigatus* was investigated in 2006 by Bhainsa and D’souza [107]. The transmission electron microscopy (TEM) micrograph showed well-dispersed and variable-sized Ag nanoparticles, most of which were spherical and only a few triangular. Compared to the intracellular biosynthesis of nanoparticles, the extracellular synthesis could be recognized as a simple but also profitable method due to the secondary stage of the bioprocess. This refers to the part where the mass of cells from stage I ends up satisfying the quality and purity requirements of the biomass.

Although the method is simple and leads to biocompatible materials, it has several drawbacks, including the difficulties in controlling the size and morphologies of the NPs, the long periods of time necessary for bioreduction, as well as the possible presence of endotoxins.

## 3. The Types of Bio-Nanosensors Used in the Food Industry

Food contamination with pathogenic microorganisms or associated toxins can cause acute and chronic diseases and can lead to epidemics and death. Pathogenic bacteria exist in a variety of shapes and types and could be highly heat resistant (e.g., *Clostridium botulinum*, *C. perfringens*, *Bacillus subtilis*, *B. cereus*), could be capable of producing heat-resistant toxins (e.g., *Staphylococcus aureus*, *Clostridium botulinum*), or could grow under temperatures of less than 10 °C or in refrigerated conditions [108]. Due to the diversity of foodborne pathogenic agents and their rapid spread, recent studies reported advances in the development of nanosensor-based technologies, such as nanobiosensors, DNA biosensors, smartphone-based biosensors [109].

**Figure 3:**
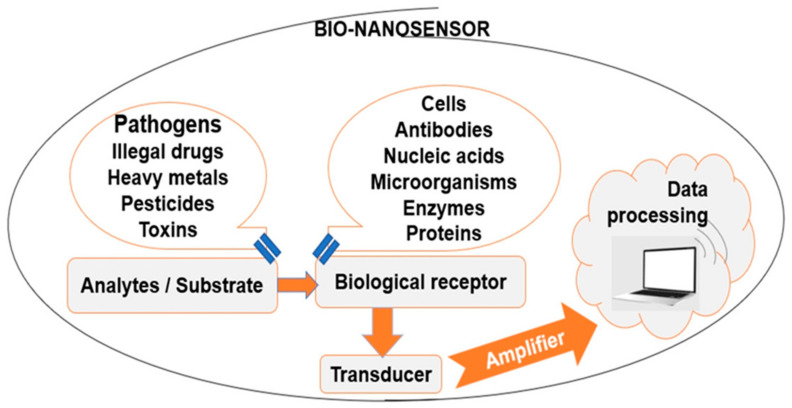
Typical bio-nanosensor components.

Food nanotechnology can be applied in order to increase food production, as well as for the development of food with higher nutritional value and quality. However, despite the numerous recent research works in this area, there still exist numerous challenges and opportunities to improve the current sensor technology. Sensing food pathogen development will be useful for quality control; consumers will know that products are compliant, and the frequency of food-borne diseases will be reduced. A typical biosensor (Figure 3) contains bio-recognition elements, such as enzymes, antibodies, proteins, cells, the analyte, such as toxins, bacteria, enzymes, antibodies, proteins, cells, and a transducer, able to convert biological response into measurable electronic signals. Digital electronic signals are directly related to the target biomolecules’ concentration [110]. 

**Figure 4:**
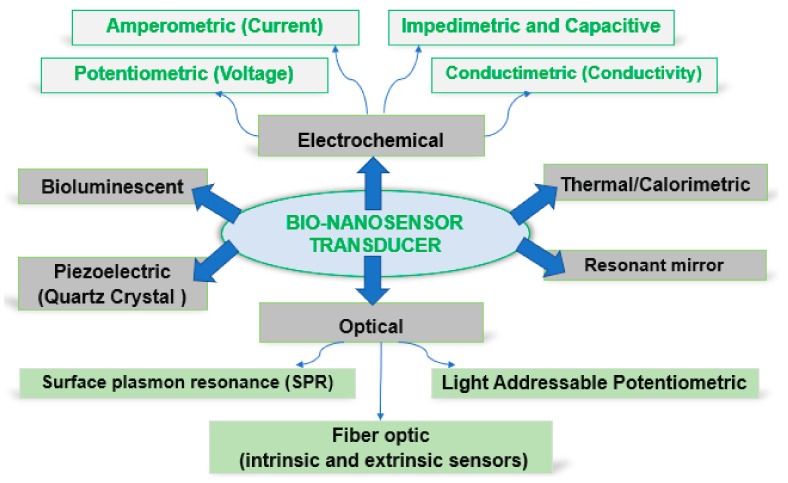
Schematic representation of biosensor technologies.

As a function of the transducer type, biosensors can be classified as in Figure 4.

Therefore, nanosensors are used to transmit the information about nanoparticles to the macroscopic world. The development of nanomaterials in the form of nanoparticles, nanofibers, nanowires, or nanotubes has found applications as bio-detectors and bio-analyzers, being capable of very fast detection, even of a single cell of foodborne pathogens. The use of very low nanoparticle doses successfully detected foodborne infections [111]. Rapid detection can be carried out through optical sensors that work on light scattering technology, which can differentiate bacterial colonies up to the genus, species, and strain level, as in the case of *Listeria*, *Staphylococcus*, *Salmonella*, *Vibrio*, and *Escherichia coli* [112,113,114]. For specific recognition of pathogens, metal, and magnetic nanoparticles [114], polymeric [115] or inorganic semiconductors have been conjugated with biologically sensitive elements (called receptors), such as antibodies, enzymes [116], antibiotics, nucleic acids [117], lipids, tissue, or microorganisms [118,119].

## 4. Use of Metallic and Metal Oxides Nanoparticles for Sensing Food Pathogens

### 4.1. Nanoparticles Application in Food Pathogen Sensing Technologies

In the past decades, numerous research works [120,121,122] discussed the potential of nanoparticles (NPs) and their incorporation into biosensor systems in order to achieve the use of NPs in biosensor applications for the detection of food pathogens. Nanotechnology-based detection methods use metallic NPs, such as gold and silver [123], with tunable size and shape.

The metallic nanoparticles can be used as sensing platforms for the target pathogen (such as *Escherichia coli*, *Staphylococcus aureus*, *Salmonella typhymurium*, *Enterococcus faecalis*, *Aspergillus niger*, *Fusarium oxysporum*) from different food matrices, generating a combined response pattern selective for every pathogen [124,125]. The response will be quantified by different sensing platforms, such as colorimetric chemosensors. Nanostructures have the potential to be used as simple and good sensing elements in a colorimetric sensor system due to their functions of receptors and indicators simultaneously, leading to color change. The mechanism of metallic nanoparticles (leading to color change)—electrochemical–surface plasmon resonance sensor (EC-SPR)—phytosynthesized nanomaterials, can be used as coating for the sensing electrode modification for identification of the bacterial pathogens in clinical and food-related samples [16,126].

The simplest method of detection based on nanotechnology is the colorimetric detection. For example, the interaction/binding between gold nanoparticles and analytes represents an interaction that induces the aggregation of gold nanoparticles and the subsequent visual change of color from red to blue. Biodetection based on the localized surface plasmon resonance (LSPR) band modification is a method based on changing the spectral characteristics induced by a variation in the local environment of gold nanoparticles, following biospecific interactions [127]. This method is less sensitive than the aggregation tests, and since any molecule can induce a change in the LSPR band of gold nanoparticles, the method is only suitable for the detection of known analytes and only when gold nanoparticles are functionalized with molecular recognition elements, such as antibodies [128]. For the detection and identification of unknown samples, Raman spectroscopy is the most appropriate method because it is a very specific technique able to identify molecular species based on their unique Raman vibration fingerprint. The Raman signal can be drastically amplified when the analytes are adsorbed on metal surfaces, an effect known as surface-amplified Raman scattering. The surface-enhanced Raman scattering (SERS) substrate is therefore essential for efficient biodetection applications. In solution, the aggregated or anisotropic nanospheric gold nanoparticles cause the formation of hot spots between the interconnected nanoparticles that substantially amplify the Raman signal. As important as the form of gold nanoparticles is the functionalization and biocompatibility of the sensors that must allow the adsorption of the analytes for an efficient, stable, and reproducible SERS signal [129,130].

*E. coli* O157:H7 presence in food determined serious issues for human health and financial losses for food producers. Meng Xu et al. [131] developed a rapid biosensor for *E. coli* O157:H7 detection in pure culture and in food. They obtained bifunctional polymeric nanocomposites (PMNCs), which contained antibodies (ABs-rabbit anti-*E. coli* O + K polyclonal antibodies) and glucose oxidase (GOx). Firstly, magnetic beads (MBs) were linked to GOx by the streptavidin–biotin reaction, followed by obtention of a thin film of polydopamine (PDA) on MB-GOx by dopamine (DA) self-polymerization. The AuNPs were biochemically synthesized on the MBs-GOx@PDA PMNCs through the in situ reduction in chloroauric acid by the H_2_O_2_. As stated by the authors, the use of biocompatible PDA allowed the GOx to maintain its enzymatic activity to catalyze glucose to produce H_2_O_2_. ABs and GOx adsorption measurement led to ABs/GOxext/AuNPs/MBs-GOx@PDA PMNCs, which was used to separate the pathogenic agent from the food and to amplify the signal. Mixing the target bacteria capture method with labeling steps, these bifunctional PMNCs with AuNPs proved short detection time. Additionally, the transfer of the biological recognition to the signal took place because the numerous linking sites on the bacteria had abundant and active enzymes attached. Regarding the *E. coli* O157:H7 capture efficiency, the PMNCs with AuNPs captured about 89% bacteria cells between 10^2^ and 10^5^ CFU/mL. Considering about 10% bacteria losses, the PMNCs proved a good efficacy. The properties of this prepared biosensor demonstrated its efficacy in sensing food pathogens. The AuNPs were obtained using the method previously presented by Fu et al. [132]. Fu et al. [132] developed performant polymeric bionanocomposites (PBNCs) containing PDA, Pt nanoparticles (PtNPs), GOx, AuNPs, and an antibody (antihuman immunoglobulin) by in situ synthesis of NPs. PBNCs exhibited high enzymatic activity, useful in signal improvement, and high antibody loading for efficient immuno-recognition.

Zhongyu Fu et al. [133] outlined a rapid AuNPs-based colorimetric assay for detection of *Listeria monocytogenes* and *Salmonella enterica*, using the PCR method and thiol-labeled primers (PCR being applied to amplify the *hly* gene of *L. monocytogenes* and the hut gene of *S. enterica*). AuNPs (13 nm in diameter) were prepared by the citrate reduction in HAuCl_4_ and exhibited a characteristic surface plasmon band centered at 520 nm. Mixing the products with thiol label (as PCR results) and AuNPs, the sulphur–gold linkage resulted. These are more tolerant of salt than primers linked to AuNPs, so colorimetric testing using naked eye or spectrophotometric measurements could be used for pathogenic bacteria detection in food.

Xiaolin Huang et al. [134] reported a homogeneous AuNPs-based immunoassay using dynamic light scattering (DLS) for detection of *Listeria monocytogenes* (*L. monocytogenes*) from lettuces. Having a large surface (0.5 μm × 2.0 μm), *L. monocytogenes* possess numerous antigen epitopes, which act as binding carriers for the AuNPs, leading to AuNPs-coated bacteria complexes. In their research study, various parameters were changed until optimized development conditions were established and also to separate and concentrate *L. monocytogenes* from lettuce samples. Regarding the sensitivity and reproducibility of the AuNPs-based DLS immunoassay, NPs with three different diameters were obtained and used (small, medium, and large). Small-sized AuNPs were synthesized using the Li et al. method [135], and medium and large sizes were obtained using another method [136] based on hydroquinone (HQ). When large AuNPs (100 nm) were used, the limit of detection (LOD) for *L. monocytogenes* in lettuce probes reached 2.2 × 10^1^ CFU/g. Their values were much lower than the maximum limit imposed in Canada (100 CFU/g). The procedure was applied to 17 common pathogenic bacteria, but the results clearly demonstrated that the best results were recorded for *L. monocytogenes* detection.

Miranda et al. [137] developed a sensitive colorimetric enzyme-AuNPs sensing for detection of bacteria. The AuNPs functionalized with quaternary ammonium ligands are electrostatically bound to β-galactosidase (β-Gal), leading to the inhibition of enzymatic activity without denaturation. Upon exposure to the bacteria, the functionalized NPs bind to their anionic surface, releasing the enzyme, which turns the pale-yellow substrate to red. This method demonstrated, in the case of *E. coli* (XL1), a visual sensitivity of 1 × 10^4^ bacteria/mL, but for other bacteria, the sensitivity may vary depending on the bacteria species.

In recent years, NPs-based LSPR biosensors have been studied for their efficiency in the recognition of bacteria [138]. In the case of some LSPR biosensors with a low detection limit, the incorporation of AuNPs and an aptamer was studied. In 2017, Seo Yeong Oh et al. [139] proposed a portable plasmonic biosensor for *Salmonella typhimurium* detection in artificially contaminated pork meat. Large-area AuNPs were immobilized on the glass substrate, and the aptamer was bound to the AuNPs by a dipping process. Firstly, AuNPs with controlled size were synthesized as Neus G. Bastus et al. described [140]. Secondly, a glass substrate treated for impurities was removed and then covered with 0.5% 3-aminopropyl)-triethoxysilane (APTES) as a linker with the amine group, which was prepared. Finally, the amino-functionalized substrate was immersed into an AuNPs solution for 8–9 h, and the change from colorless to burgundy demonstrated the adhesion of AuNPs onto the glass substrate. The obtained LSPR sensing devices exhibited a detection limit of 10^4^ CFU/mL in pure culture and also in artificially contaminated pork meat probes, the *S. typhimurium* being identified using an aptamer as a linker between the AuNPs-conjugated system and bacteria.

A highly sensitive and rapid method for colorimetric detection of *Bacillus cereus* (*B. cereus*) in milk was developed by coupling asymmetric PCR (producing long genomic DNA fragments from the cereulide synthetase gene, *cesB*) with propidium monoazide (PMA) and unmodified AuNPs [141]. Under optimum conditions, the LOD for viable emetic *B. cereus* in phosphate-buffered saline (PBS) and milk was 9.2 × 10^1^ CFU/mL and 3.4 × 10^2^ CFU/mL, respectively. According to the results, this method was appropriate to respect the maximum limit established by the Commission Regulation (EC) No 2073/2005 (500 CFU/mL) and is considered a good detection test for food-borne pathogens.

The simultaneous detections of *Shigella boydii* (*S. boydii*) and *Escherichia coli* O157:H7 (*E. coli* O157:H7) in three types of food products using the colloidal gold immunochromatographic strip (ICS) was reported [142]. The pathogen agent detection from food products was evaluated by strip tests and the ELISA method. The LOD by the strip tests reached 10^6^ CFU/mL for both food-borne pathogens, without any cross-reaction with other related bacteria. The LOD was enhanced to 4 CFU/mL when all three food products containing *S. boydii* and *E. coli* O157:H7 were pre-incubated.

In 2013, Yun Ju Sung et al. [143] prepared a sensing mechanism for *Staphylococcus aureus* detection in milk. The resulting sensing method based on a composite antibody/AuNPs/carboxylated magnetic nanoparticle demonstrated high capture efficiencies for *S. aureus* in PBS of 96% and 78% in milk. Using the properties of both the magnetic nanoparticle and the AuNPs, such as magnetic separation and signal generation, the LOD of this colorimetric sensor in PBS for *S. aureus* reached 1.5 × 10^3^ CFU and 1.5 × 10^5^ CFU in milk.

A variety of sensing devices are also based on silver NPs (AgNPs) [144,145]; this type of NPs presents some disadvantages, such as NPs degradation leading to Ag^+^ ions caused by functionalization and oxidation phenomenon appearing on the surface of AgNPs. A very efficient immunosensor for *S. aureus* detection was fabricated by Abdolkarim Abbaspouret et al. [146]. For a system assay in the form of a sandwich, the following components were used: a biotinylated anti-*S. aureus* primary aptamer fixed on magnetic beads (MB) that were coated with streptavidin for sample capture, an anti-*S. aureus* secondary aptamer conjugated with AgNPs [147,148] for increasing signal, and implicit for target bacteria detection, and an electrochemical stripping voltammetry read-out for sensitive detection. The obtained immunosensor exhibited a dynamic range from 10 to 1 × 10^6^ CFU/mL and a LOD of 1.0 CFU/mL.

The *Escherichia coli* detection with surface-enhanced Raman scattering (SERS) was achieved by Naja et al. [149] using polyclonal antibodies on protein-A-modified AgNPs. In their experimental data, *Escherichia coli* ATCC 13529 and *Rhodococcus rhodochrous* ATCC 17895 (American Type Culture Collection, Manassas, VA, USA) were used, and the results showed that, although the two bacteria were present, only *E. coli* was absorbed by the AgNPs. The proposed Raman scattering technique and also attaching an appropriate polyclonal antibody to the target bacteria on the nanoparticles surface assured specific and more selective bacteria detection results as compared to the traditional Raman spectroscopy. SERS with silver nano substrates were used for detection and identification of three food-borne pathogens (*E. coli* O157: H7, *S. aureus,* and *Salmonella*) investigated by Wei et al. [150]. For the silver colloidal NPs synthesis, the microwave heating method was applied. The rapid SERS procedure [151] combined with silver colloidal nanoparticles, which play an important role in the signal enhancement for SERS, proved high reproducibility for all three studied food-borne pathogens. In 2013, Cowcher et al. [152] used SERS for rapid and sensitive detection of *Bacillus* in food, mixing silver colloidal nanoparticles with the bacteria. The use of SERS techniques [153] for food-borne pathogen detection in food is constantly evolving. This led to the use of different types of metal nanoparticles (MNPs) and metal oxides (MOs) [154], such as copper oxide (CuO), titanium dioxide (TiO_2_), Zinc oxide (ZnO), or silver oxide (Ag_2_O), due to their promising properties in food industries. CuNPs [155] can be used to detect pathogenic agents responsible for food spoilage. Being less expensive, Cu and copper oxide (CuO) are more attractive compared to noble metals. TiO_2_ nanocrystals, prepared as D. C. Pan et al. described [156], were used as optical nanomaterials for spectroscopic detection of *Salmonella* in milk [157]. A polyclonal antibody-conjugated magnetic nanoparticles complex captured *Salmonella* bacteria under an external magnetic field. The resulting magnetic nanoparticle/*Salmonella* was re-dispersed in PBS and exposed to antibody-immobilized TiO_2_NPs, exhibiting an increase in light absorption. Combining the immuno-magnetic separation with optical nanomaterials for *Salmonella* detection, a TiO_2_NPs-based immunoassay was obtained as a good alternative to the traditional methods based on PCR. The limit of detection for the targeted bacterium in milk was higher than 100 CFU/mL. When ZnO-NPs with special electronic configuration are used, the actions of the released Zn^2+^ ions are very important. The contact of ZnO-NPs with the pathogen surface determined the formation of electrostatic forces affecting the cell membrane [16]. Some examples of metallic nanoparticles applied in sensing food pathogens are presented in Table 1.

### 4.2. Functionalization of Metallic Nanoparticles for Sensing Food Pathogens

The development of successful bio-sensors for food pathogens sensing systems, as can be seen from the examples provided in the previous paragraphs, can be achieved by several different approaches.

a. Direct interaction of the nanoparticles and the analytes, inducing a visible color change and a corresponding spectral LSPR change. This is the simplest approach, in which the colloidal NPs solution is mixed with a solution containing the analyte. The spectral change can be easily followed by UV-Vis spectrometry. The approach was presented in several studies, being used for the detection of *Listeria monocytogenes* and *Salmonella enterica* (as demonstrated by Zhongyu Fu et al. [133]) or for the detection of emetic *B. cereus* [141] and *Bacillus* spores [152] in milk samples.
b. Physical deposition of NPs on different substrates represents another viable alternative. It can be achieved either by direct synthesis on the surface of different materials (as demonstrated by Xu et al. [131] and Fu et al. [132]) or by the deposition of the NPs on substrates (for example, on glass substrates coated with (3-aminopropyl)-triethoxysilane), as presented by Oh et al. [139].
c. The coating of metallic nanoparticles with specific antibodies was also presented in several studies. For example, Huang et al. [134] obtained anti-*Listeria monocytogenes* mAbs-coated AuNPs by adding the antibodies solution to the AuNPs solution, under magnetic stirring, further blocked with polyethylene glycol and bovine serum albumin; using the same method, anti-clenbuterol monoclonal antibody/AuNPs (further deposited on paper strips) [135] or double monoclonal antibodies (against *Shigella boydii* and *Escherichia coli* O157:H7) conjugated gold nanoparticles (also deposited on test strips) were obtained.
d. The functionalization of metallic nanoparticles was presented in several works. For example, Miranda et al. [137] obtained cationic AuNPs by obtaining pentanethiol-coated AuNPs (using a two-phase synthesis method), which were further quaternary-ammonium functionalized by the Murray place-exchange method and further deposited on test strips. Magnetic nanoparticles were functionalized with glutaraldehyde (in order to form amine groups and amine-reactive crosslinkers on the NPs surfaces), on which monoclonal *Salmonella* antibodies were further immobilized [157].

All the approaches presented have their particular advantages and disadvantages. However, in order to pass from the laboratory scale to practical day-by-day application, the biosensor needs to be simple to use and to reveal the presence of the food contaminants. One such approach is represented by the incorporation of biopolymers to develop smart packaging materials [175]. This would allow the development of easy-to-use sensing technologies, which would provide a visual indicator for the final consumer. Although this approach is very promising, there remains the issue of nanoparticle migration into the products, which raises health issues and remains to be addressed via toxicity studies.

## 5. Phytosynthesized Nanoparticles as Biosensors

In the large number of studies regarding the sensor application of metallic nanoparticles, the use of phytosynthesized NPs can be encountered as an alternative, “green chemistry” approach, usually for the detection of other types of contaminants (and not food pathogens). Although not directly focused on the subject of the present review paper, these applications can provide a glimpse on the future perspectives of pathogen detection technologies.

One such example is represented by the detection of H_2_O_2_, a known oxidizing and bleaching agent used in several industries (including pulp and paper, food, or pharmaceutical industry). As its discharge can cause significant damage to the aquatic ecosystems, there is a need for its rapid and reliable detection. Among other approaches, the use of phytosynthesized nanoparticles was evaluated for the biosensing of H_2_O_2_. Krishnaraj et al. [176] phytosynthesized different types of organic/inorganic nanoparticles and their composites. The best results were obtained for a chitosan/silver composite, with a reduction peak current of 43 ± 1.4 μA and a detection limit and sensitivity of 50 nM and 542 μA/mM×cm^2^, respectively. Similar approaches were used by different authors, applying phytosynthesized nanoparticles using algae or plant parts for hydrogen peroxide detection, heavy metals, organic substances (including pesticides or pharmaceuticals), or even for the determination of a biomarker of prostate cancer [177] (Table 2).

Generally, the experimental procedure can follow two pathways: a colorimetric methodology, in which the nanoparticles-containing solutions are mixed with solutions containing the targeted analyte and the color changes and/or changes in the UV-vis spectra are recorded, respectively, or a more complex methodology, in which electrodes (usually glassy carbon electrode) are changed directly by the nanoparticles or nanoparticles-containing composites.

All the examples presented in Table 2 show a very good selectivity, reproducibility, and sensitivity along low limits of detection and quantification when compared with the currently applied detection methods.

## 6. Safety and Regulatory Issue

Particular attention is given to the use of metal nanoparticles that can be successfully implemented in numerous domains, such as food, biotechnology, medicine, cosmetology, etc. [203,204]. However, there are several risks associated with both the production and application of nanoparticles, with possible consequences for the environment and human health alike. A lot of studies evidenced that NPs, having a small cell size and cellular organs, present very good mobility, both in living organisms and in the environment, being able to penetrate biological structures, affecting their normal functioning [205,206].

The risks related to the use of nanoparticles can be avoided by testing changes in morphological properties, the degree of inhibition of cell population growth and development, changes in the biochemical activity of living organisms and cellular components. Yeasts, algae, and other microorganisms can be used as a study model in microbial biotechnology. For functional tests to measure the inhibition or stimulation degree of a compound, which is observed by the maximum effect of 50% (dose/response curve), it is suggested to use the terms of effective concentration (EC50%) or inhibition concentration (IC50%) by the compound. The most widely used indicators for assessing toxicity are lethal concentration 50 (LC50) and maximum permissible concentration (CMT). Thus, these tests provide the opportunity to perform comprehensive research of the nanoparticles’ effect on the body [207,208].

The above indicators determine the opportunity to study the characteristics, properties, and effects of nanoparticles designed to elucidate the degree of influence on living organisms, knowledge that can promote ideas for their efficient use.

The global and European organizations [209] take precautions in providing rules for assessing the risks of nanomaterials.

In August 2021, the European Food Safety Authority published two guidelines in their updated versions—“Guidance on risk assessment of nanomaterials to be applied in the food and feed chain: human and animal health” and “Guidance on technical requirements for regulated food and feed product applications to establish the presence of small particles including nanoparticles” [210,211]. Additionally, the Food and Drug Administration (FDA) has issued a Guidance for Industry—“Considering whether an FDA-regulated product involves the application of nanotechnology” [212]—which represents the agency’s opinion on this subject.

Being best related to MNPs use in the food industry, the European legislation can be found in the following:

(a) Regulation (EC) No 1935/2004 refers to the materials and articles intended to encounter food [213].
(b) Commission Regulation (EC) No 450/2009 acts on the active and intelligent materials and articles that will interact with food, defined as “materials and articles which monitor the condition of packaged food or the environment surrounding the food” [214].
(c) Commission Regulation (EU) No 10/2011 refers to the plastic materials and articles intended that will interact with food and emphasizes that “substances in nanoform” could have various toxicological properties [215].

Regulation (EC) No 178/2002 is a General Food Law Regulation and lays down the general principles and requirements of food law [216].

## 7. Conclusions and Future Perspectives

As can be seen from the example provided in the present review paper, nanomaterials in general and metallic/metal oxides nanoparticles in particular can provide important instruments for the identification and even quantification of food pathogens. Considering the potential risks to consumer’s health, this subject should be one of continuous development in the search for safer edibles.

In order to develop any type of application using nanomaterials, the synthesis methods should be carefully selected, considering all their particular advantages and disadvantages.

The incorporation of nanoparticles in sensing techniques is usually performed by several methods. The simplest approach is the direct contact of the nanoparticle solution with the analyte; this colorimetric assay is based on the SPR peak displacement when the pathogen is present, providing a faster, easier-to-use, and equally reliable method (although often with higher sensitivity) compared with other currently applied methods. Other approaches are represented by the deposition of NPs on substrates or polymeric materials, antibodies’ coatings, or surface functionalization of NPs and their deposition on test strips.

Future works in this area should be focused on the development of ready-to-use devices, either in the form of test strips or as smart packaging materials (although the latter solution could raise some concerns regarding the NPs leaching and their potential toxicity).

Considering the safety issues previously presented, the phytosynthesized nanoparticles, with their tremendous advantages (related especially to lower toxicity but also to an increased antimicrobial effect compared with other types of synthesized NPs) [37,83], can slowly find their well-deserved place. Using phytosynthesized nanoparticles, the contribution of the phytoconstituents to the final application can be harvested. As presented in Chapter 5, the phytosynthesized nanoparticles are currently applied in a series of biosensing assays, although not currently focused on food pathogen detection. This can help develop a completely new field of research: phytosynthesized NPs for food applications. As is the case for other applications involving phytosynthesized NPs, these technologies present several bottlenecks, including the successful stabilization of the NPs and development of easy-to-use assays, viable over a long period of time, technology up-scaling (most of the presented technologies being validated only at the laboratory level) toward higher technology readiness levels, as well as a more profound action required for the implementation of “green chemistry” principles and a shift in nanomaterials development toward more ecological alternatives, as well as to a superior use of vegetal wastes. Additionally, the development of sensing devices based on phytosynthesized nanoparticles of specific metals (i.e., Ag) raises several issues regarding the protection from oxidation of the NPs. This could be overcome, for example, by designing a technology for the application of metallic oxide nanoparticles.

## Figures and Tables

**Table 1:** Food-borne pathogen, predominant symptoms, and the metallic nanoparticles used for sensing food pathogens.

Target of Assay	Predominant Symptoms	Type of NP-Based Biosensor	Biosensing Element	Detection Limit	Ref.
*E. coli*	Diarrhea, abdominal pain, nausea, vomiting	AuNPs (flower-shaped, F-AuNPs and sphere-shaped, S-AuNPs)	In situ reaction between NPs and specific primer (target gene *wzy*)	50 pg/μL *E. coli* 0157:H7	[158]
*E. coli*	Diarrhea, abdominal pain, nausea, vomiting	AuNPs	Aptamers against ethanolamine and *E. coli* O111:B4 lipopolysaccharides	1 µg/mL *E. coli*	[159]
*Listeria monocytogenes*	Headache, fever, chills	AuNPs (flower-shaped, F-AuNPs and sphere-shaped, S-AuNPs)	In situ reaction between NPs and specific primer (target gene *hly*)	10 pg/μL;	[160]
*Listeria monocytogenes*	Headache, fever, chills	Phytosynthesized flower-shaped AuNPs	NPs hybridized with primers (target genes *hlyA*F and *hlyA*R)	100.4 ng	[161]
*Salmonella enterica*	Fever, abdominal cramps, diarrhea, vomiting	AuNPs	Biotinylated rabbit anti-*Salmonella* polyclonal antibody	10^4^ CFU/mL in PBS and 10^5^ CFU/mL in milk	[158]
*Salmonella typhimurium*	Fever, abdominal cramps, diarrhea, vomiting	AuNPs (flower-shaped, F-AuNPs and sphere-shaped, S-AuNPs)	In situ reaction between NPs and specific primer (target gene *hut*)	10 pg/μL	[160]
*Salmonella typhimurium*	Fever, abdominal cramps, diarrhea, vomiting	AgNPs	Cationic AgNPs functionalized with anti-*Salmonella* antibody	10^2^ cells/mL	[162]
*Bacillus cereus*	The emetic and diarrheal syndrome, abdominal cramps	AuNPs	AuNP-based colorimetric assay combined with asPCR amplification and propidium monoazide treatment	9.2 × 10^1^ CFU/mL in 0.01 M phosphate-buffered saline and 3.4 × 10^2^ CFU/mL in milk	[141]
*Campylobacter jejuni*	Fever, arthralgia, chills, headache,	Gold-Palladium nanoparticles (Au@Pd)	NPs covered with specific DNA aptamer ()	100 CFU/mL	[163]
*Staphylococcus aureus*	Nausea, abdominal pain, vomiting, diarrhea,	Gold and iron oxide (Fe_3_O_4_/Au) nanoparticles	Etching-enhanced peroxidase-like catalytic activity of gold nanoparticles	10 CFU/mL	[164]
*Shigella* spp.	Fever, abdominal cramps, diarrhea, vomiting	AuNPs	Aptamer coated NPs	80 CFU/mL *Shigella flexneri*	[165]
			Aptamer surface functionalized composite material containing an Eu-complex and AuNPs	10 CFU/mL *Shigella sonnei*	[166]
*Yersinia enterocolitica O:8 strains*	Abdominal pain, diarrhea, fever	AuNPs	Monoclonal antibody labeled AuNP	5 CFU/mL in milk and pork samples	[167]
*Trichinella spiralis*	Gastroenteritis, fever, muscular pain	AuNPs	anti-rabbit polyclonal antibody conjugated AuNPs	-	[168]
*Clostridium botulinum*	Respiratory paralysis, double or blurred vision, loss of light reflex	AuNPs	Immobilization of cleaved SNAptide with cysteine ends onto AuNPs via the thiol group	0.25 ng/mL	[169,170]
*Staphylococcus aureus*	Nausea, abdominal pain, vomiting, diarrhea,	AuNPs	Aptamer (anti *S. aureus* immunoglobulin Y) modified NPs	10 FCU/mL	[147]
*Pseudomonas aeruginosa*	The emetic and diarrheal syndrome, abdominal cramps	AuNPs	Aptamer (*P. aeruginosa*-specific aptamer F23) modified NPs	60 CFU/mL	[171]
*Aspergillus niger*	Asthma or other chronic lung diseases	AuNPs	AuNPs conjugated with thiol-containing fungal spore-binding peptide ligands	∼>50 spores	[172]
*Candida*	Oral thrush, pseudomembranous, erythematous (atrophic) and hyperplastic	Fe_3_O_4_NPs, AgNPs	Fe_3_O_4_NPs/polyethylenimine composites captured *Candida,* while positively charged AgNPs were used as SERS substrate	-	[173,174]

**Table 2:** Biosensing using phytosynthesized nanoparticles *.

NP Type	Phytosynthesis Plants	Biosensing Target and Procedure	Results	Ref.
SeNP, AgNPs	*Cucurbita pepo* L. leaves	Detection of H_2_O_2_ using glassy carbon electrode coated with NPs	Reduction peak current: 14 ± 0.5/37 ± 1.3 μA	[176]
FeNPs	*Ipomoea pes-tigridis* L. leaves	Detection of H_2_O_2_ using glassy carbon electrode coated with FeNPs/reduced graphene oxide composites	Linearity: 0.1 μM–2.15 mM. LOD: 0.056 μM. Sensitivity: 0.2085 μA/mM × cm^2^. Selectivity: determinations in the presence of dopamine, uric acid, ascorbic acid, catechol, and glucose	[178]
ZnONPs	*Corymbia citriodora* (Hook.) K.D. Hill and L.A.S. Johnson leaves	Detection of H_2_O_2_ using glassy carbon electrode coated with NPs	Linearity: 0.1–150 μM. LOD: 0.07 μM. Selectivity: determinations in the presence of uric acid, ascorbic acid, and glucose	[179]
AgNPs	*Euphorbia hirta* L. leaves	Colorimetric detection of H_2_O_2_	LOD: 10^−7^ M	[180]
AgNPs	*Cassia fistula* L. -phenolic-rich extract	Colorimetric detection of H_2_O_2_	Linearity: 10–200 μM. LOD: 3.0 μM	[181]
AgNPs	*Garcinia mangostana* L. fruits	Detection of Hg(II) in the range 1–50 μM	LOD/LOQ: 2.6 μM/8.9 μM	[182]
AuNPs	*Cistanche deserticola* Ma	Colorimetric detection of Pb(II) using NPs stabilized on poly(styrene-co-maleic anhydride)	Linearity: 0–100 μM. LOD: 0.03 μM. Selectivity: determinations in the presence of Fe^2+^, Cu^2+^, Mg^2+^, Zn^2+^, Cr^3+^, Al^3+^, Cd^2+^, Mn^2+^	[183]
AuNPs	*Annona muricata* L. fruit pulp	Colorimetric detection of Cd (II)/paper-based sensors	Linearity: 0.045–0.18 µM and 0.22–8.90 µM. LOD: 1.13 × 10^−10^/4.45 × 10^−8^ M. Selectivity: determinations in the presence of Al^3+^, Ba^2+^, Co^2+^, Fe^2+^, Hg^2+^, In^2+^, K^+^, Li^+^, Mg^2+^, Mn^2+^, Na^+^, Ni^2+^, Pb^2+^, Pt^2+^, Sn^2+^, Zn^2+^	[184]
CuNPs	*Juglans regia* L. green husk	Colorimetric detection of Hg(II)	LOD: 10 mM. Selectivity: determinations in the presence of K^+^, Ca^2+^, Pb^2+^	[185]
AgNPs	*Persea americana* Mill. peel	Colorimetric detection of Al(II) and Cr(II)	LOD: 0.04/0.05 mg/kg. Selectivity: determinations in the presence of Ni(II), Cd(II), Al(III), Hg(II), Cr(III), Ba(II), Pb(II), Zn(II), Co(II), Mn(II), Cu(II), Ca(II), Mg(II), and K(I)	[186]
Ag/AgClNPs	*Syzygium cumini* (L.) Skeels. fruits	Colorimetric detection of clindamycin and Fe^3+^	Linearity: 10.0–100.0 µM. LOD: 1.2 µM. R^2^ = 0.99 (clindamycin). Linearity: 10.0–350.0 µM. LOD: 5.6 µM (for Fe^3+^)	[187]
AgNPS	*Rumex hastatus* D. Don roots	Colorimetric detection of Cu(II)	Linearity: 1–90 µM. LOD: 0.26 µM. Selectivity: determinations in the presence of Na^+^, K^+^, Mg^2+^, Ca^2+^, Ba^2+^, Fe^2+^, Ni^2+^, Pb^2+^, Sn^2+^, Hg^2+^, Zn^2+^	[188]
AgNPs	*Camellia sinensis*(L.) Kuntze leaves	Colorimetric detection of Fe(III)	Linearity: 1–25 μM. LOD = 0.532 μM. LOQ= 1.77 μM. Reproducibility (RSD) = 1.49%.	[189]
AgNPs	Dry root of *Hedysarum polybotrys* Hand.-Mazz.*-Radix Hedysari*	Colorimetric detection of Fe(II)	LOD: 1.5 μM. Linearity: 10 μM–500 μM. Selectivity: determinations in the presence of Ag^+^, Cu^2+^, Zn^2+^, Pb^2+^, Ni^2+^, Na^+^, Cr^3+^, Fe^3+^, K^+^.	[190]
AgNPs	*Moringa oleifera* Lam. flower	Colorimetric detection of Cu(IV)	Linearity: 1–12 mM. Sensitivity: 0.249/mM.	[191]
AgNPs	*Sonchus arvensis* L. leaves	Colorimetric detection of Fe(III) and Hg(II)	LOD: 10^−3^ M. Selectivity: determinations in the presence of Li^+^, Al^3+^, Cr^3+^, Mn^2+^, Fe^3+^, Co^2+^, Ni^2+^, Cu^2+^, Hg^2+^, Cd^2+^, Pb^2+^_._	[192]
AuNPs	*Fragaria vesca* L. leaves	Detection of uric/ascorbic acids using glassy carbon electrode coated with NPs	Linearity: 0.1−0.98, 0.98−190/1–10, 10–5750 µM. LOD: 0.16/0.05 µM. LOQ: 0.49/0.15 µM. Sensitivity: 0.617, 0.169/0.130, 0.050 μA/μM	[193]
AuNPs, AgNPs	*Calendula officinalis* L. flowers	Prostatic specific antigen detection using quince seed mucilage/NPs composite	Linearity: 0.1 pg/mL–100 ng/mL. LOD: 0.087 pg/mL	[177]
AgNPs	*Cyanthillium cinereum* (Carl Linnaeus) H. Rob leaves	Dopamine sensing (0.01 mM–0.1 mM), using carbon paste electrodes modified with AgNP	Oxidation peak with peak potentials at 0.366 V, 0.998 correlation coefficient	[194]
CuNPs	*Ocimum tenuiflorum* L. leaves	Glucose sensing using glassy carbon electrode coated with NPs	Sensitivity: 1065.21 μA/mM × cm^2^. Response time: < 3 s. Linear range: 1–7.2 mM. LOD: 0.038 μM.	[195]
ZnONPs	*Ocimum tenuiflorum* L. leaves	Glucose sensing using glassy carbon electrode coated with NPs	Linear range: 1–8.6 mM. LOD: 0.046 μM. Sensitivity: 681.60 μAm/M × cm^2^	[196]
AuNPs	*Bischofia javanica* Blume leaves	Chloramphenicol determination in milk, powdered milk, honey, and eye drops using glassy carbon electrode coated with NPs decorated graphene oxide film	Linearity: 1.5–2.95 μM. LOD: 0.25 μM. Sensitivity: 3.81 μA/μM × cm^2^	[197]
CuNPs	*Moringa oleifera* Lam	Detection of daptomycin and meropenem using NPs deposited on the surface of screen-printed carbon electrodes	LOD: 0.01 g/L.	[198]
NiFe_2_O_4_ NPs	*Ixora coccinea* L. leaves	Determination of pentachlorophenol using glassy carbon electrode chemically modified with NPs	Linearity: 0.01–90 μM at pulse amplitude of 80 mV/s. LOD/LOQ: 0.0016/0.005 μM. Selectivity: determinations in the presence of Cu^+2^, Ca^+2^, Mg^+2^, K^+^, Cl^−^, SO_3_^−2^, trichlorophenol, ascorbic acid, hydroquinone, endosulfan, carbofuran	[199]
AgNPs	*Araucaria angustifolia* (Bertol.) Kuntze nuts	Detection of paracetamol using glassy carbon electrode coated NPs and exfoliated graphite nanoplatelets composites	Repeatability/reproducibility: 1.8%/4.0%. Linearity: 4.98 × 10^−6^–3.38 × 10^−5^ mol/L. LOD: 8.50 × 10^−8^ mol/L.	[200]
Ag-AuNPs	*Citrus × sinensis* (L.) Osbeck peels	Detection of caffeine using a platinum electrode modified with polypyrrole and NPs	LOD: 2.02 μM. Sensitivity: 0.75 μA/μM. Linearity: 0–59 μM. Selectivity: determinations in the presence of ascorbic acid, dopamine, glucose, fructose, and common ions (K^+^, Cl^−^, Na^+^, and NO_3_^−^)	[201]
Ag_2_ONPs	*Brassica rapa* L. Pekinensis Group leaves	Detection of p-nitrophenol using a carbon black/nickel foam–NP electrode	Linearity: 0.1 pM–1 mM. Response time: 5 s. LOD: 0.7 pM.	[202]
SeNP, AgNPs	*Cucurbita pepo* L. leaves	Detection of H_2_O_2_ using glassy carbon electrode coated with NPs	Reduction peak current: 14 ± 0.5/37 ± 1.3 μA	[176]
FeNPs	*Ipomoea pes-tigridis* L. leaves	Detection of H_2_O_2_ using glassy carbon electrode coated with FeNPs/reduced graphene oxide composites	Linearity: 0.1 μM–2.15 mM. LOD: 0.056 μM. Sensitivity: 0.2085 μA/mM × cm^2^. Selectivity: determinations in the presence of dopamine, uric acid, ascorbic acid, catechol, and glucose.	[178]
ZnONPs	*Corymbia citriodora* (Hook.) K.D. Hill and L.A.S. Johnson leaves	Detection of H_2_O_2_ using glassy carbon electrode coated with NPs	Linearity: 0.1–150 μM. LOD: 0.07 μM. Selectivity: determinations in the presence of uric acid, ascorbic acid, and glucose.	[179]

* LOD—limit of detection; LOQ—limit of quantification; NP—nanoparticles.

## Data Availability

Not applicable.

## Acronyms and Abbreviations

Au	Gold
Ag	Silver
WHO	World Health Organization
QD	quantum dots
ASTM	American Society for Testing and Materials
NP	nanoparticle
mm	millimeter
nm	nanometer
NM	nanomaterial
HPLC-MS	High-performance liquid chromatography–mass spectrometry
PCR	Polymerase chain reaction
ELISA	Enzyme-linked immunosorbent assay
Pt	Platinum
Pd	Palladium
Zn	Zinc
Cd	Cadmium
Cu	Copper
Fe	Iron
Ni	Nickel
Co	Cobalt
HAuCl_4_	Tetrachloroauric Acid
H_2_PtCl_6_	Hexachloroplatinic acid
RhCl_3_	Rhodium (III) chloride
PdCl_2_	Palladium(II) chloride
R_min_	The minimum race
cm	centimeter
TiO_2_	Titanium dioxide
RF	radio frequency
K	Kelvin
kHz	kilohertz
MHz	megahertz
kW	kilowatt
MW	megawatt
atm	atmosphere
sec	seconds
N	Nitrogen
DMF	dimethylformamide
PEG	polyethylene glycol
UV	ultraviolet
AuNPs	gold nanoparticles
°C	degrees Celsius
min	minutes
Mn_3_O_4_	Manganese (II,III) oxide
ZnO	Zinc oxide
SnO_2_	Tin oxide
PbO	Lead (II) oxide
CVD	Chemical vapor deposition
aq	aqueous
TEM	Transmission electron microscopy
EC-SPR	Electrochemical–surface plasmon resonance sensor
DNA	Deoxyribonucleic Acid
LSPR	Localized surface plasmon resonance
SERS	Surface-enhanced Raman scattering
*E. coli*	*Escherichia coli*
PMNCs	polymeric nanocomposites
antibodies	ABs
GOx	glucose oxidase
MBs	magnetic beads
PDA	polydopamine
DA	dopamine
CFU	colony-forming unit
mL	milliliter
PtNPs	platinum nanoparticles
PBNCs	polymeric bionanocomposites
DLS	Dynamic light scattering
*L. monocytogenes*	*Listeria monocytogenes*
μm	micrometer
IMS	Immunomagnetic separation method
HQ	hydroquinone
LOD	Limit of detection
g	gram
β-Gal	β-galactosidase
*S. typhimurium*	*Salmonella typhimurium*
APTES	3-aminopropyl)-triethoxysilane
h	hours
PBS	phosphate-buffered saline
EC	Commission Regulation
No	Number
*S. boydii*	*Shigella boydii*
ICS	immunochromatographic strip
*S. aureus*	*Staphylococcus aureus*
ATCC	American Type Culture Collection
MNPs	metal nanoparticles
MOs	metal oxides
CuO	copper oxide
Ag_2_O	silver oxide
CuNPs	Copper nanoparticles
TNs	Titanium dioxide nanocrystals
pg	picograms
F-AuNPs	flower-shaped gold nanoparticles
S-AuNPs	sphere-shaped gold nanoparticles
Fe_3_O_4_	Iron oxide
SeNP	Selenium nanoparticle
FeNP	Iron nanoparticle
kg	kilogram
K	Potassium
Mg	Magnesium
Ca	Calcium
Ba	Barium
Sn	Tin
Hg	Mercury
Cr	Chromium
Al	Aluminum
EC	Effective concentration
IC	inhibition concentration
LC	lethal concentration
CMT	maximum permissible concentration
FDA	Food and Drug Administration
